# Rational application of the first‐line chemotherapy and immune checkpoint inhibitors in advanced nonsmall cell lung cancer: A meta‐analysis

**DOI:** 10.1002/cam4.2407

**Published:** 2019-07-11

**Authors:** Rui Cao, Jie‐Tao Ma, Shu‐Ling Zhang, Li Sun, Yang Liu, Xiang‐Yan Zhang, Wei Jing, Le‐Tian Huang, Cheng‐Bo Han

**Affiliations:** ^1^ Department of Oncology Shengjing Hospital of China Medical University Shenyang China

**Keywords:** chemotherapy, immunotherapy, meta‐analysis, nonsmall cell lung cancer, programmed death‐ligand 1

## Abstract

**Objective:**

To compare the relative efficacy of immune checkpoint inhibitors (ICIs) or chemotherapy (CT) alone, or their combination modality in the first‐line treatment of advanced nonsmall cell lung cancer (NSCLC).

**Methods:**

This meta‐analysis was performed on the eligible randomized controlled trials (RCTs) after searching web databases and meeting abstracts. The main research endpoints were the comparisons of median overall survival (mOS), the OS rate of 6 months (OSR6m), 1 year (OSR1y) and 2 years (OSR2y), median progression‐free survival (mPFS), the PFS rate of 6 months (PFSR6m) and 1‐year (PFSR1y), objective response rates (ORR), and treatment‐related adverse events (TRAEs).

**Results:**

Eleven RCTs comprising 6278 cases were included. In the subgroup of programmed death‐ligand 1 (PD‐L1) ≥50%, compared with chemotherapy, the ICIs showed similar OSR6m (*P* > 0.05), but significantly improved efficacy in mOS, OSR1y, OSR2y, and ORR (all *P* < 0.05), also had less grade ≥ 3 TRAEs. Compared with pembrolizumab alone, pembrolizumab plus CT in the subgroup of PD‐L1 ≥ 50% had similar mOS, OSR6m, OSR1y, and PFSR1y (all *P* > 0.05), but significantly improved mPFS, PFSR6m, and ORR (all *P* < 0.05 for interaction). Compared with the CT group, ICIs plus CT group with PD‐L1 ≥ 50% or <1% showed significant benefit in OS, PFS, and ORR (all *P* < 0.05). However, in the ICIs plus CT group with 1% ≤ PD‐L1 ≤ 49%, only PFS and ORR showed significant benefit compared with CT group (all *P* < 0.05), but not for results of OS.

**Conclusions:**

The findings support the rationale for using pembrolizumab alone in the first‐line treatment of PD‐L1 ≥ 50% advanced NSCLC due to the similar OS and lower grade ≥ 3 TRAEs. However, the combination of ICIs and chemotherapy is strongly recommended in patients with PD‐L1 ≤ 49% for significant survival benefit.

## 1 INTRODUCTION

Lung cancer is the leading cause of cancer‐related death worldwide,^1^ and nonsmall cell lung cancer (NSCLC) accounts for 80%–85% of lung cancer cases, two‐thirds of which are unresectable due to initial diagnosis of advanced disease.^2^ The development of target therapy has greatly improved the survival of the patients who have variations in targets such as epidermal growth factor receptor (EGFR) and anaplastic lymphoma kinase (ALK).^3^ Nevertheless, approximately one‐half patients of NSCLC have no specific driver gene mutations.^4^ Platinum‐based doublet chemotherapy is the standard first‐line therapy for these patients. However, it has limited curative effect and sometimes patients could not confront the side effects of chemotherapy.

Immune checkpoint inhibitors (ICIs) that can block immunosuppressive molecules such as programmed cell death‐protein 1 (PD‐1) or its ligand PD‐L1, and cytotoxic T‐lymphocyte associated protein 4 have been proven to be effective in treating various kinds of cancers by restoring antitumor immunity. ICIs that have been clinically applied to NSCLC include pembrolizumab, nivolumab, atezolizumab, durvalumab, and ipilimumab.^5^, ^6^, ^7^, ^8^, ^9^, ^10^ Several prospective studies have demonstrated that both ICIs monotherapy and combination regimens showed encouraging efficacies in the treatment of NSCLC.^11^, ^12^ An early meta‐analysis conducted by Wang et al provided clinical evidence that either ICI monotherapy or in combination with chemotherapy improved survival in patients with advanced NSCLC, and ICI group had fewer adverse events (AEs).^13^ However, the meta‐analysis included all lines of treatment, and was not focused on the first‐line treatment. In addition, several recent randomized controlled trials (RCTs) have reported the latest results and showed more evidence of immunotherapy for the first‐line treatment of NSCLC. Nevertheless, there is still considerable controversy about how to choose the best first‐line treatment, that is, ICIs or chemotherapy alone or in combination. Currently, several studies have shown that PD‐L1 or tumor mutational burden (TMB) status can be applied to evaluate the efficacy and survival of immunotherapy, so it is promising to use these biomarkers to make therapeutic decisions.^14^, ^15^ Herein, a meta‐analysis was performed to make a contrast in terms of the efficacy and safety between ICIs in combination with chemotherapy and chemotherapy or ICIs alone in the first‐line treatment of advanced NSCLC.

## 2 MATERIALS AND METHODS

### 2.1 Search strategy

Two investigators independently made a comprehensive search of Cochrane Library, EMBASE, PubMed, and Web of Science databases with the following keywords: (atezolizumab OR durvalumab OR ipilimumab OR nivolumab OR pembrolizumab) AND (NSCLC OR lung cancer). The final literature search was performed on 15 December 2018. In addition to computer search, manual searches were conducted for the abstracts from conferences.

### 2.2 Study selection

Studies that met the following inclusion criteria were included. They are as follows: (a) Research type and eligible patients: phase II or III RCTs designed for patients with histologically or cytologically confirmed stage IIIB or IV or recurrent metastatic NSCLC, who had chemotherapy‐naive, no sensitizing EGFR or ALK alteration. (b) Intervention measure: patients were randomly assigned to experimental group and control group. The experimental groups included ICIs (nivolumab or pembrolizumab or atezolizumab or durvalumab or ipilimumab) or ICIs in combination with chemotherapy or other ICIs. The control group was chemotherapy (platinum‐based chemotherapy or combined with antiangiogenic therapy). Only the drug of ICIs differed between the two groups. (c) Research outcome: the primary endpoints included one of the following: median progression‐free survival (mPFS), median overall survival (mOS), or objective response rate (ORR).

The exclusion criteria included non‐RCTs, systematic reviews, case reports, and repeated published studies.

### 2.3 Quality assessment and data extraction

The Cochrane Collaboration guidelines were used in evaluating the risk of bias. The characteristics of each trial were extracted, including pathological type, the status of PD‐L1 and TMB, etc The endpoints were also extracted, including the mPFS, mOS, ORR, and grade ≥ 3 treatment‐related adverse events (TRAEs). The OS rate (OSR) of 6 months (OSR6m), and 1 year (OSR1y) and 2 years (OSR2y), and the PFS rate (PFSR) of 6 months (PFSR6m) and 1 year (PFSR1y) were extracted from Kaplan‐Meier curves using Engauge Digitizer v.10.8 software (produced by Mark Mitchell 2014; https://github.com/markummitchell/engauge-digitizer) to further analyze the impact on short‐term and long‐term survival.

### 2.4 Statistical analysis

This meta‐analysis was performed using Stata software version 12.0 (StataCorp, College Station). The ORR, OSR6m, OSR1y, OSR2y, PFSR 6m, PFSR1y, and the rate of grade ≥ 3 TRAEs were expressed as risk ratios (RRs), and the mPFS and mOS were expressed as hazard ratios (HRs). Meta regression with fixed effect models was employed to assess the potential effects of clinical variables on outcomes, unless *I* ^2^ was more than 50%, a random effect model was used in which case. We firstly evaluated the clinical efficacy and toxicity between ICIs and chemotherapy in selected cases (PD‐L1 ≥ 50% or high TMB) and between ICIs combined with chemotherapy and chemotherapy in unselected cases, respectively. Then, the efficacy of ICIs in combination with chemotherapy vs chemotherapy was further analyzed according to the expression level of PD‐L1. The heterogeneity of efficacy was evaluated by an interaction test^16^ between ICIs alone and in combination with chemotherapy, between pembrolizumab plus chemotherapy and atezolizumab plus chemotherapy, and between nonsquamous and squamous cell carcinoma.

## 3 RESULTS

### 3.1 Characteristics of the studies included

**Figure 1:**
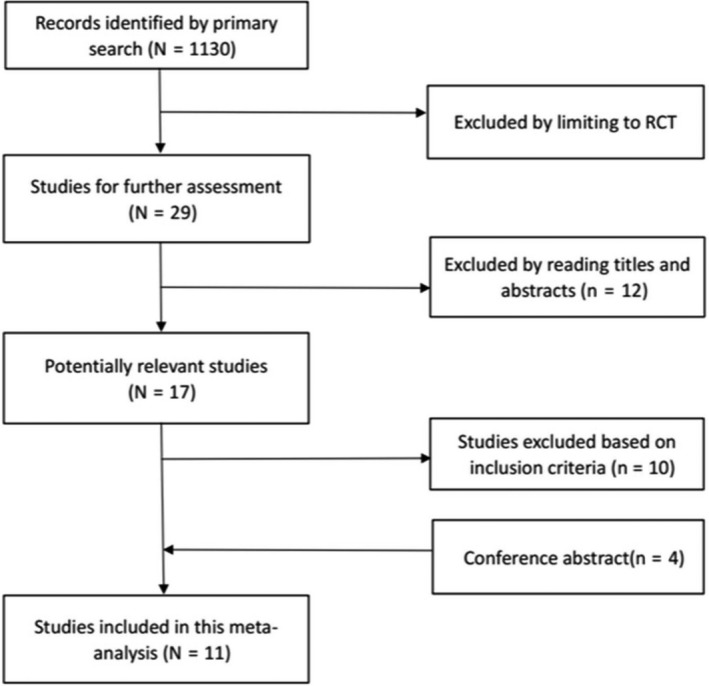
Overview of study search and selection

The characteristics of the included studies are shown in Table 1. According to the inclusion and exclusion criteria, 11 RCTs comprising 6,278 NSCLC patients were enrolled in this meta‐analysis (Figure 1). Four of these studies involved comparisons between ICIs and chemotherapy^17^, ^18^, ^19^, ^20^ and seven involved comparisons between ICIs combined with chemotherapy and chemotherapy.^21^, ^22^, ^23^, ^24^, ^25^, ^26^, ^27^ ICIs were compared with standard chemotherapy in selected NSCLC (PD‐L1 > 50% or high TMB).

**Table 1:** The characteristics of the studies included

Study	Author/published year	Histologies	Status of PD‐L1	Randomized arms	Case number	Stratified by PD‐L1	Case number	Status of TMB
KN‐024	Martin 2016	Nonsqu./Squ.	≥50%	Pembrolizumab	154	TPS ≥ 50%	154	
				Platinum‐based chemo.	151	TPS ≥ 50%	151	
KN‐042	Gilberto 2018	Nonsqu./Squ.	≥1%	Pembrolizumab	637	TPS ≥ 50%	299	
						TPS 1%‐49%	338	
				Platinum‐based chemo.	637	TPS ≥ 50%	300	
						TPS 1%‐49%	337	
KN‐407	Luis 2018	Squ.	Any	Pembrolizumab, CBP, PTX or nab‐PTX	278	TC3	73	
						TC1/TC2	103	
						TC0	95	
				Placebo, CBP, PTX or nab‐PTX	281	TC3	73	
						TC1/TC2	104	
						TC0	99	
KN‐021G	Langer 2016	Nonsqu.	Any	Pembrolizumab, pemetrexed and CBP	60	NA	NA	
				Pemetrexed and CBP	63	NA	NA	
KN‐189	Leena 2018	Nonsqu.	Any	Pembrolizumab, pemetrexed and CBP	410	TC3	132	
						TC1/TC2	128	
						TC0	127	
				Placebo, pemetrexed and CBP	206	TC3	70	
						TC1/TC2	58	
						TC0	63	
CM‐026	Carbone 2017	Nonsqu./Squ.	≥5%	Nivolumab	47	TPS ≥ 5%	47	≥243 mut*
				Platinum‐based chemo.	60	TPS ≥ 5%	60	
CM‐227	Hellmann 2018	Nonsqu./Squ.	Any	Nivolumab and ipilimumab	139	NA	NA	≥10 mut/Mb
			Any	Histology‐based chemo.	160	NA	NA	
			<1%	Nivolumab and histology‐based chemo.	177	TPS < 1%	177	
			<1%	Histology‐based chemo.	186	TPS <1%	186	
IMP‐150	Reck 2017	Nonsqu.	Any	Atezolizumab, CBP, PTX and BEV	356	TC3 or IC3	71	
						TC1/TC2 or IC1/IC2	121	
						TC0 and IC0	167	
				CBP, PTX and BEV	336	TC3 or IC3	65	
						TC1/TC2 or IC1/IC2	105	
						TC0 and IC0	172	
IMP‐131	Robert 2018	Squ.	Any	Atezolizumab, CBP and nab‐PTX	343	TC3 or IC3	53	
						TC1/TC2 or IC1/IC2	129	
						TC0 and IC0	160	
				CBP and nab‐PTX	340	TC3 or IC3	48	
						TC1/TC2 or IC1/IC2	121	
						TC0 and IC0	171	
IMP‐132	Vassiliki 2018	Nonsqu.	Any	Atezolizumab, CBP or CDDP, and pemetrexed	292	TC3 or IC3	25	
						TC1/TC2 or IC1/IC2	63	
						TC0 and IC0	88	
				CBP or CDDP, and pemetrexed	286	TC3 or IC3	20	
						TC1/TC2 or IC1/IC2	73	
						TC0 and IC0	75	
IMP‐130	Federico 2018	Nonsqu.	Any	Atezolizumab and CBP and nab‐PTX	451	TC3 or IC3	88	
						TC1/TC2 or IC1/IC2	128	
						TC0 and IC0	235	
				CBP and nab‐PTX	228	TC3 or IC3	42	
						TC1/TC2 or IC1/IC2	65	
						TC0 and IC0	121	

Abbreviations: * mut, mutation via whole exome sequencing (WES); BEV, bevacizumab; CBP, carboplatin; CDDP, cisplatin; CM, CHECKMATE; IC0, PD‐L1 < 1% of tumor infiltrating immune cells; IC1/IC2, 1% ≤ PD‐L1 < 10% of tumor infiltrating immune cells; IC3, PD‐L1 ≥ 10% of tumor infiltrating immune cells; IMP, IMPOWER; KN, KEYNOTE; mut/Mb, mutation/megabase; NA, not available; nab‐PTX, nab‐paclitaxel; Nonsqu., nonsquamous carcinoma; PD‐L1, programmed death‐ligand 1; PTX, paclitaxel; Squ., squamous carcinoma; TC0, PD‐L1 < 1% of tumor cells; TC1/TC2, 1% ≤ PD‐L1 < 50% of tumor cells; TC3, PD‐L1 ≥ 50% of tumor cells; TMB, tumor mutational burden; TPS, tumor proportion score.

### 3.2 Quality assessment and publication bias

Table 2 summarizes the quality assessment of the included trials. No substantial asymmetry was found in the visual inspection of the funnel plots (Figure S1). Consistently, the Egger linear regression test and Begg rank correlation test also found no evidence of publication bias.

**Table 2:** The quality assessment of the randomized controlled trials included

Study	Author/published year	Random sequence generation (selection bias)	Allocation concealment (selection bias)	Blinding of participant and personnel (performance bias)	Blinding of outcome assessment (detection bias)	Incomplete outcome data (attrition bias)	Selective reporting (reporting bias)	Other bias*
KN‐024	Martin 2016	Low risk	Unclear risk	High risk	Low risk	Low risk	Low risk	Low risk
KN‐042	Gilberto 2018	Low risk	Unclear risk	High risk	Low risk	Low risk	Low risk	Low risk
KN‐407	Luis 2018	Low risk	Unclear risk	Low risk	Low risk	Low risk	Low risk	Low risk
KN‐021G	Langer 2016	Low risk	Low risk	High risk	Low risk	Low risk	Low risk	Low risk
KN‐189	Leena 2018	Low risk	Unclear risk	Low risk	Low risk	Low risk	Low risk	Low risk
CM‐026	Carbone 2017	Low risk	Unclear risk	High risk	Low risk	Low risk	Low risk	Low risk
CM‐227	Hellmann 2018	Low risk	Unclear risk	High risk	Low risk	Low risk	Low risk	Low risk
IMP‐150	Reck 2017	Low risk	Unclear risk	High risk	High risk	Low risk	Low risk	Low risk
IMP‐131	Robert 2018	Low risk	Unclear risk	High risk	High risk	Low risk	Low risk	Low risk
IMP‐132	Vassiliki 2018	Low risk	Unclear risk	High risk	High risk	Low risk	Low risk	Low risk
IMP‐130	Federico 2018	Low risk	Unclear risk	High risk	High risk	Low risk	Low risk	Low risk

Note:

CM, CHECKMATE; IMP, IMPOWER; KN, KEYNOTE.

^*^Other bias refers to other issues that lead to high risk of bias.

### 3.3 Comparisons between ICIs and chemotherapy in selected NSCLC with PD‐L1 ≥ 50% or high TMB

Four RCTs compared the efficacy and safety between ICIs and chemotherapy, including 1310 patients with high PD‐L1 expression defined as tumor proportion score (TPS) ≥50%, who were treated with pembrolizumab, or with high TMB defined as TMB ≥ 10 mutation/megabase (mut/Mb) or TMB ≥ 243 mutations in whole exome sequencing, who were treated with nivolumab monotherapy or combined with ipilimumab.

#### 3.3.1 PD‐L1 ≥ 50% for pembrolizumab

**Figure 2:**
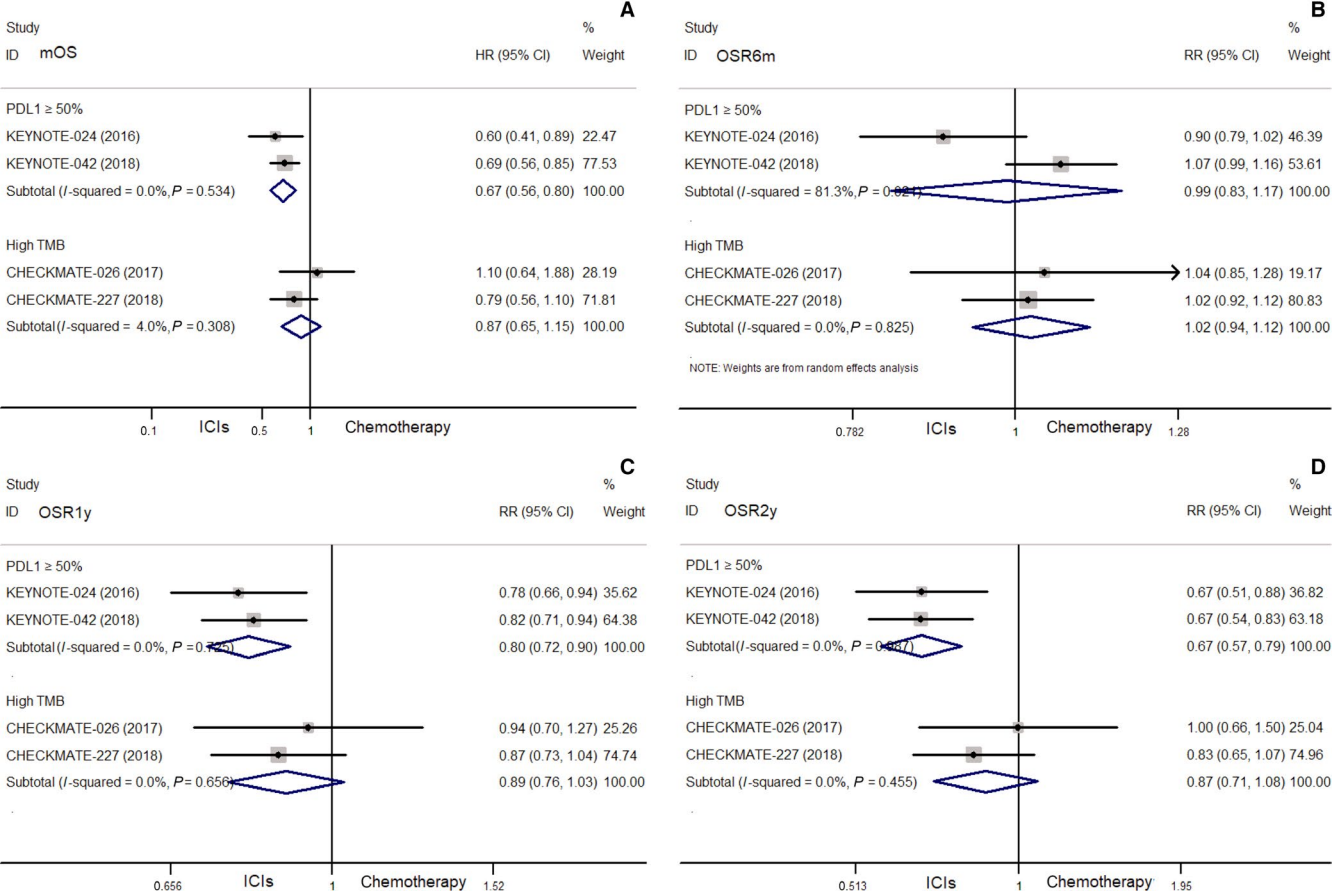
Comparisons of median overall survival (mOS) (A), the OS rates of 6 months (OSR6m) (B), 1 year (OSR1y) (C) and 2‐years (OSR2y) (D) between ICIs and chemotherapy in selected nonsmall cell lung cell lung cancer (NSCLC) (programmed death‐ligand 1 (PD‐L1) ≥50% or high tumor mutational burden (TMB)

ICIs showed significantly benefit in mOS (HR = 0.67, 95% CI: 0.56‐0.80), OSR1y (RR = 0.80, 95% CI: 0.72‐0.90), and OSR2y (RR = 0.67, 95% CI: 0.57‐0.79) compared with chemotherapy (all *P* < 0.001). The OSR6m showed no obvious difference between the two groups (RR = 0.99, 95% CI: 0.83‐1.17, *P* = 0.877) (Figure 2).

**Figure 3:**
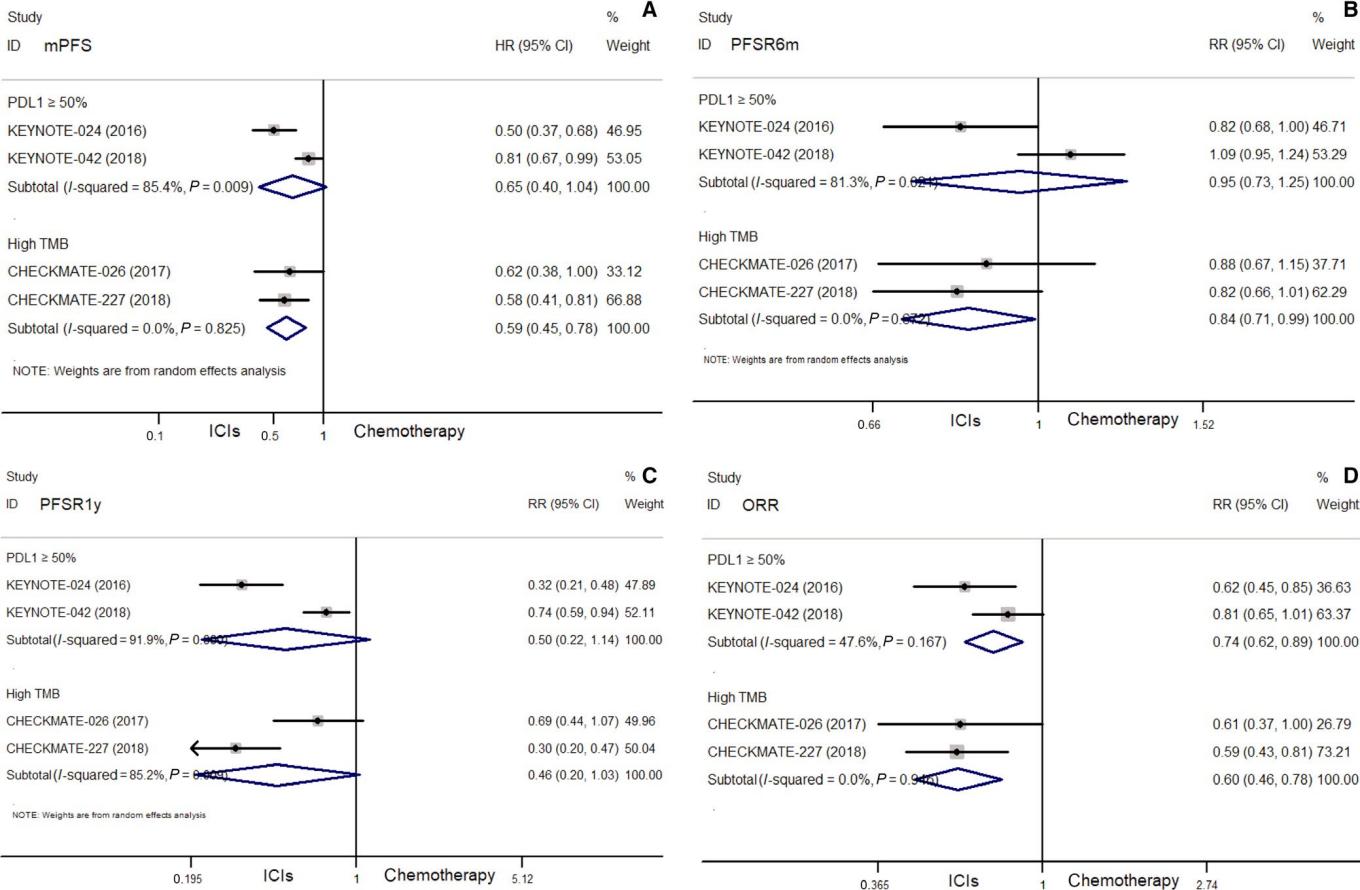
Comparisons of median progression‐free survival (mPFS) (A), the PFS rate of 6 months (PFSR6m) (B) and 1 year (PFSR1y) (C), objective response rates (ORR) (D) between ICIs and chemotherapy in selected NSCLC (PD‐L1 ≥ 50% or high TMB)

ICIs showed no obvious difference in mPFS (HR = 0.65, 95% CI: 0.40‐1.04, *P* = 0.069), PFSR6m (RR = 0.95, 95% CI: 0.73‐1.25, *P* = 0.729), and PFSR1y (RR = 0.50, 95% CI: 0.22‐1.14, *P* = 0.098) compared with chemotherapy. However, ICIs showed significant benefit in ORR (RR = 0.74, 95% CI: 0.62‐0.89, *P* = 0.001) vs chemotherapy (Figure 3).

ICIs showed less toxicity in grade ≥ 3 TRAEs in comparison with chemotherapy (RR = 0.45, 95% CI: 0.38‐0.53, *P* < 0.001) (Figure S2A).

#### 3.3.2 High TMB for nivolumab monotherapy or combined with ipilimumab

Compared with chemotherapy, nivolumab monotherapy or combined with ipilimumab showed no obvious difference in terms of mOS (HR = 1.10, 95% CI: 0.64‐1.88; 0.79, 95% CI: 0.56‐1.10, respectively), OSR6m (RR = 1.04, 95% CI: 0.85‐1.28; 1.02, 95% CI: 0.92‐1.12, respectively), OSR1y (RR = 0.94, 95% CI: 0.70‐1.27; 0.87, 95% CI: 0.73‐1.04, respectively), and OSR2y (RR = 1.00, 95% CI: 0.66‐1.50; 0.83, 95% CI: 0.65‐1.07, respectively) (all *P*> 0.05) (Figure 2). While nivolumab combined with ipilimumab showed benefit in terms of mPFS (HR = 0.58, 95% CI: 0.41‐0.81), PFSR1y (RR = 0.30, 95% CI: 0.20‐0.47) and ORR (RR = 0.59, 95% CI: 0.43‐0.81) (all *P* < 0.05), and a trend toward significance of PFSR6m (RR = 0.82, 95% CI: 0.66‐1.01) compared with chemotherapy (Figure 3).

Nivolumab monotherapy showed less toxicity in grade ≥ 3 TRAEs than chemotherapy (RR: 0.35; 95% CI: 0.26‐0.46), while nivolumab combined with ipilimumab showed no obvious difference with chemotherapy (RR = 0.86; 95% CI: 0.73‐1.02) (Figure S2A).

### 3.4 Comparisons between combination of chemotherapy with ICIs and chemotherapy alone in unselected NSCLC

Seven RCTs including 3930 patients compared ICIs combined with chemotherapy vs chemotherapy in advanced NSCLC with any expression of PD‐L1.

**Figure 4:**
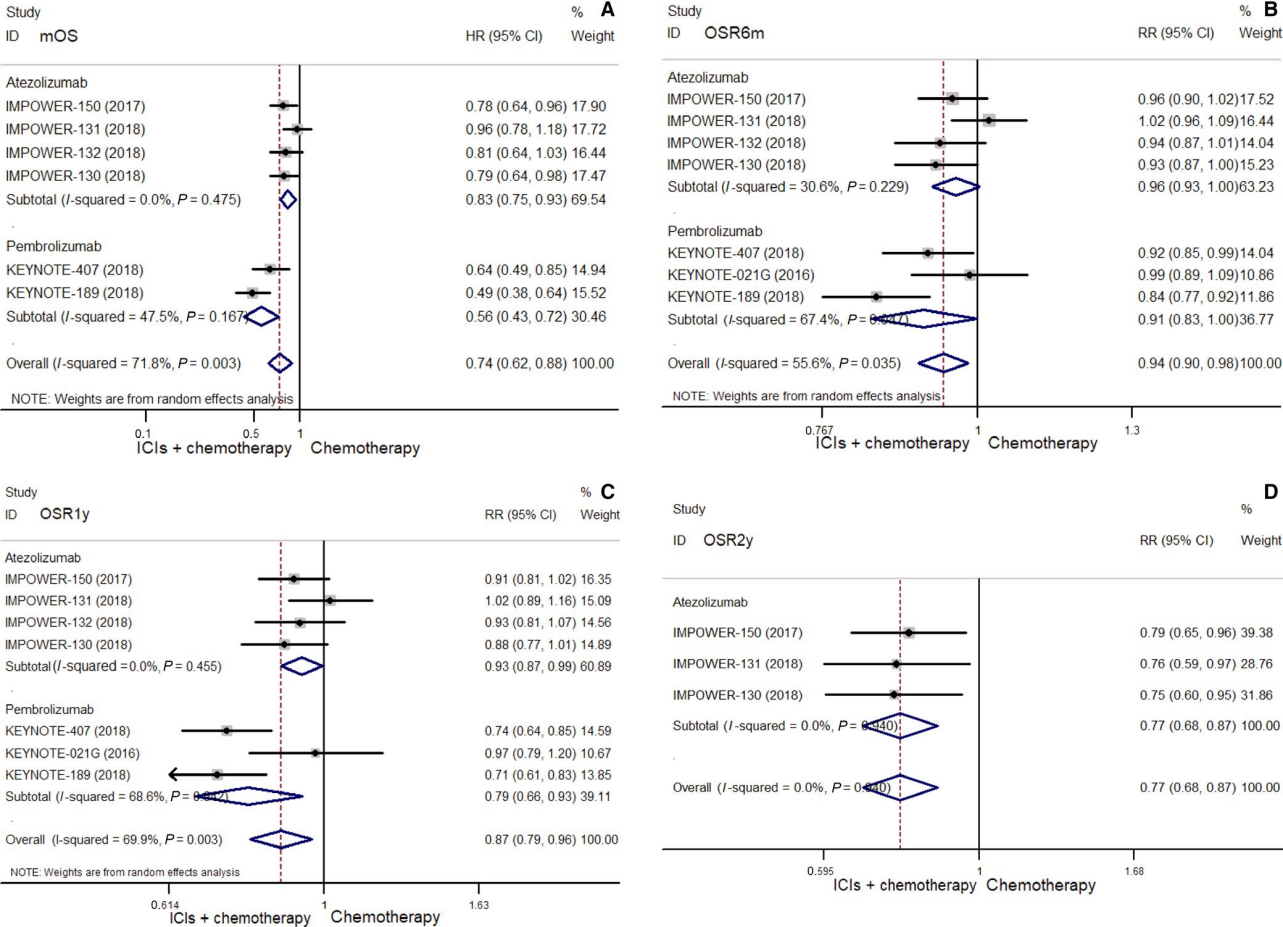
Comparisons of mOS (A), OSR6m (B), OSR1y (C) and OSR2y (D) between combination of chemotherapy with ICIs and chemotherapy alone in unselected NSCLC

**Figure 5:**
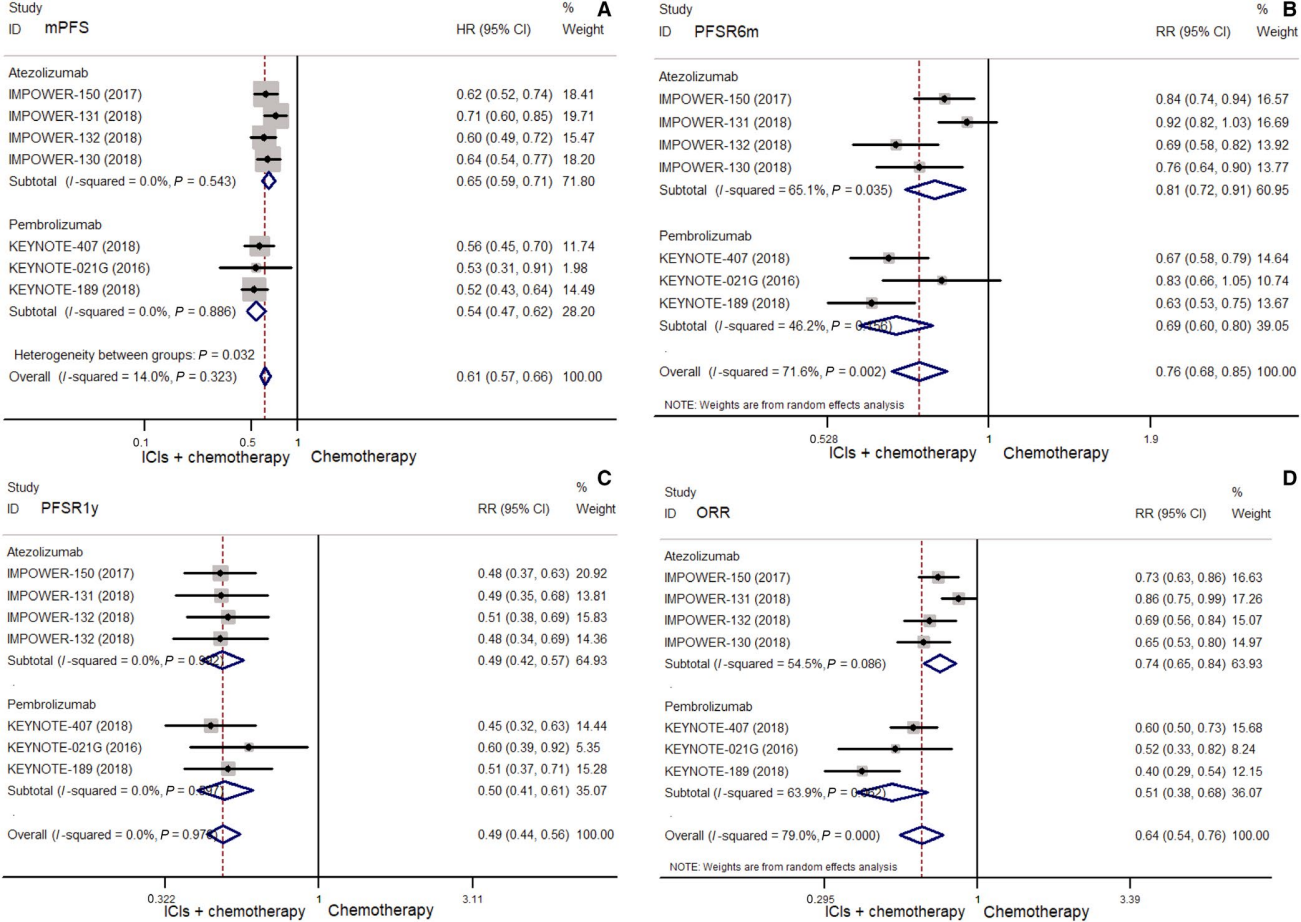
Comparisons of mPFS (A), PFSR6m (B) and PFSR1y (C), ORR (D) between combination of chemotherapy with ICIs and chemotherapy alone in unselected NSCLC

Compared with chemotherapy, ICIs combined with chemotherapy indicated significantly benefit in mOS (HR = 0.74, 95% CI: 0.62‐0.88), OSR6m (RR = 0.94, 95% CI: 0.90‐0.98), OSR1y (RR = 0.87, 95% CI: 0.79‐0.96), OSR2y (RR = 0.77, 95% CI: 0.68‐0.87) (all *P* < 0.01) (Figure 4), mPFS (HR = 0.61, 95% CI: 0.57‐0.66), PFSR6m (RR = 0.76, 95% CI: 0.68‐0.85), PFSR1y (RR = 0.49, 95% CI: 0.44‐0.56), and ORR (RR = 0.64, 95% CI: 0.54‐0.76) (Figure 5) (all *P* < 0.001).

ICIs combined with chemotherapy indicated more toxicity in terms of grade ≥ 3 TRAEs than chemotherapy (RR = 1.16, 95% CI: 1.06‐1.26, *P* = 0.001) (Figure S2B).

### 3.5 Comparisons between pembrolizumab plus chemotherapy and atezolizumab plus chemotherapy in unselected NSCLC

Compared with atezolizumab plus chemotherapy, pembrolizumab plus chemotherapy showed significant benefit in mOS, mPFS, and ORR (*P* = 0.006, 0.029, and 0.022 for interaction, respectively).

### 3.6 Comparisons between nonsquamous and squamous cell carcinoma treated with ICIs and chemotherapy

Chemotherapy combined with either atezolizumab or pembrolizumab showed similar benefit in mOS (*P* = 0.115 and 0.163 for interaction, respectively) and mPFS (*P* = 0.191 and 0.619 for interaction, respectively) between nonsquamous and squamous cell carcinoma, while patients with nonsquamous cell carcinoma showed significantly increased ORR (*P* = 0.021 and 0.033 for interaction, respectively) compared with patients with squamous cell carcinoma (Figures S3A, S4A, S4D).

### 3.7 Comparisons between combination of chemotherapy with ICIs and chemotherapy alone in selected NSCLC

#### 3.7.1 PD‐L1 ≥ 50% of tumor cells (TC3) or PD‐L1 ≥ 10% of tumor infiltrating immune cells (IC3)

Six RCTs including 760 patients compared ICIs combined with chemotherapy vs chemotherapy in advanced NSCLC with TC3 for pembrolizumab or TC3/IC3 for atezolizumab.

ICIs combined with chemotherapy showed a significant benefit in mOS (HR = 0.61, 95% CI: 0.49‐0.77), OSR6m (RR = 0.88, 95% CI: 0.82‐0.95), OSR1y (RR = 0.77, 95% CI: 0.68‐0.87) (all *P* < 0.001), and OSR2y (RR = 0.73, 95% CI: 0.57‐0.94, *P* = 0.014) compared with chemotherapy (Figure S5). Additionally, ICIs combined with chemotherapy showed benefit in terms of mPFS (HR = 0.41, 95% CI: 0.34‐0.49), PFSR6m (RR = 0.69, 95% CI: 0.55‐0.86), PFSR1y (RR = 0.40, 95% CI: 0.30‐0.53), and ORR (RR = 0.59, 95% CI: 0.42‐0.81) (all *P* < 0.001) (Figure S6).

#### 3.7.2 1% ≤ PD‐L1 < 50% of TC (TC1/TC2) or 1% ≤ PD‐L1 < 10% of IC (IC1/IC2)

A total of six RCTs comprising 1198 patients compared ICIs combined with chemotherapy vs chemotherapy in advanced NSCLC with TC1/TC2 for pembrolizumab and TC1/TC2 or IC1/IC2 for atezolizumab.

Compared with chemotherapy, ICIs combined with chemotherapy showed no obvious difference in mOS (HR = 0.77, 95% CI: 0.55‐1.07), OSR6m (RR = 0.98, 95% CI: 0.93‐1.03), OSR1y (RR = 0.87, 95% CI: 0.74‐1.04), and OSR2y (RR = 0.94, 95% CI: 0.69‐1.30) (all *P*> 0.05) (Figure S7). Sensitivity analyses showed that IMPOWER‐131 has a significant effect on the results. When removed, combination group showed significant benefit in terms of mOS (HR = 0.65, 95% CI: 0.50‐0.79) and OSR1y (RR = 0.82, 95% CI: 0.72‐0.93), and trend toward significance of OSR6m (RR = 0.95, 95% CI: 0.90‐1.01) and OSR2y (RR = 0.81, 95% CI: 0.64‐1.02).

Furthermore, combination therapy showed benefit in mPFS (HR = 0.63, 95% CI: 0.55‐0.72), PFSR6m (RR = 0.80, 95% CI: 0.72‐0.88), PFSR1y (RR = 0.50, 95% CI: 0.40‐0.63) (all *P* < 0.001), and ORR (RR = 0.74, 95% CI: 0.57‐0.97, *P* = 0.032) (Figure S8).

#### 3.7.3 PD‐L1 < 1% of TC (TC0) and IC (IC0)

Seven RCTs including 1936 patients compared ICIs combined with chemotherapy vs chemotherapy in advanced NSCLC with TC0 for pembrolizumab and nivolumab, or TC0/IC0 for atezolizumab.

Except OSR6m, combination therapy showed significantly benefit in terms of mOS (RR = 0.78, 95% CI: 0.67‐0.90, *P* = 0.001), OSR1y (HR = 0.87, 95% CI: 0.76‐0.99, *P* = 0.040), and OSR2y (HR = 0.70, 95% CI: 0.58‐0.85, *P* < 0.001) compared with chemotherapy. The OSR6m showed no obvious difference between two groups (RR = 0.96; 95% CI: 0.91‐1.01, *P* = 0.105) (Figure S9).

Similarly, combination therapy showed benefit in mPFS (HR = 0.72, 95% CI: 0.65‐0.80), PFSR6m (RR = 0.86, 95% CI: 0.80‐0.94), PFSR1y (RR = 0.61, 95% CI: 0.50‐0.73) (all *P* < 0.001), and ORR (RR = 0.70, 95% CI: 0.56‐0.88, *P* = 0.002) (Figure S10).

### 3.8 Comparisons between pembrolizumab alone and in combination with chemotherapy in selected NSCLC with PD‐L1 ≥ 50% or 1% ≤ PD‐L1 ≤ 49%

#### 3.8.1 PD‐L1 ≥ 50%

Compared with pembrolizumab alone, pembrolizumab combined with chemotherapy in the subgroup of PD‐L1 ≥ 50% had the similar efficacies in mOS, OSR6m, OSR1y, and PFSR1y (*P* = 0.184, 0.117, 0.351, and 0.498 for interaction, respectively) (Figures S11, S12C), but had significant benefit in mPFS, PFSR6m, and ORR (*P* = 0.038, 0.002, and 0.009 for interaction, respectively) (Figure S12A‐B and D).

#### 3.8.2 1% ≤ PD‐L1 ≤ 49%

Pembrolizumab combined with chemotherapy in the subgroup of 1% ≤ PD‐L1 ≤ 49% had significantly improved efficacies in mOS, OSR6m, and OSR1y compared with pembrolizumab alone (all *P* = 0.01 for interaction) (Figure S13).

## 4 DISCUSSION

This study enhances our understanding of the rational application of ICIs monotherapy or in combination with chemotherapy in the first‐line treatment of advanced NSCLC and thus answers several controversial questions.

One question is whether ICIs alone can improve median and long‐term overall survival (≥2 year) without increasing the risk of early death (≤6 months) in patients with high PD‐L1 expression (TPS ≥ 50%) compared with chemotherapy alone. In both KEYNOTE‐024 and KEYNOTE‐042 trials, pembrolizumab showed survival benefits in mOS and mPFS compared with chemotherapy for patients with high PD‐L1 expression. In KEYNOTE‐024, pembrolizumab also showed benefit in terms of PFSR6m (RR = 0.82, 95% CI: 0.68‐1.00, *P* = 0.047) and a trend toward significance in OSR6m (RR = 0.90, 95% CI: 0.79‐1.02, *P* = 0.089) compared with chemotherapy, by contrast, in KEYNOTE‐042, pembrolizumab showed a trend to increased early mortality (RR for OSR6m = 1.07, 95% CI: 0.99‐1.16). Our meta‐analysis indicated that for patients with TPS ≥ 50%, when compared with chemotherapy, the ICI pembrolizumab showed similar OSR6m, mPFS, PFSR6m, and PFSR1y, but benefit in terms of mOS, OSR1y, OSR2y, and ORR, and lower incidence of grade ≥ 3 TRAEs, suggesting ICIs alone obviously improved median and long‐term OS without increasing early risk of death.

The second question is whether ICIs in combination with chemotherapy can further improve survival, especially the early survival of patients with high PD‐L1 expression compared with chemotherapy or ICIs alone. Our meta‐analysis showed that in comparison with chemotherapy alone, ICIs combined with chemotherapy showed benefit in OS, PFS. and ORR for these patients with high PD‐L1 expression. Furthermore, interaction tests showed when compared with the ICI pembrolizumab alone, the ICI pembrolizumab in combination with chemotherapy had similar efficacies in terms of mOS, OSR6m, OSR1y, and PFSR1y (all *P*> 0.05 for interaction), but significantly improved efficacies in mPFS, PFSR6m, and ORR. The results suggested that combination therapy could neither improve OS nor reduce the risk of early death, so we suggest that the ICI pembrolizumab alone can be recommend as a preferred the first‐line treatment for patients with high PD‐L1 expression. This is consistent with what has been recommended by the National Comprehensive Cancer Network (NCCN) guidelines. Nevertheless, we also observed that combination therapy significantly reduced the risk of early disease progression and increased the percentage responders who otherwise do not respond to pembrolizumab alone. Clinically, if patients with a good physical condition have extensive metastases or large tumor burdens before treatment, there is an urgent need for tumor remission to reduce the tumor size and improve the symptoms of the patient, then the combination therapy can be moderately recommended. It should be noted that the interactive comparison between ICIs monotherapy and ICIs in combination with chemotherapy was merely limited to pembrolizumab and might not be extended to other ICIs. Further direct comparisons through RCTs are warranted.

The third question is whether additional ICIs to the first‐line chemotherapy is necessary for patients with low or negative expression for PD‐L1. Previous studies have shown that the synergistic activity and acceptable safety profile could be observed by the combination of checkpoint inhibitors and chemotherapy in the first‐line treatment of advanced NSCLC.^28^, ^29^ With the inclusion of two recent studies, our study drew consistent conclusion that compared with standard chemotherapy, the combination of chemotherapy and ICIs, regardless of atezolizumab or pembrolizumab could acquire significant benefits in mOS, OSR1y, mPFS, PFSR6m, PFSR1y, and ORR for PD‐L1 unselected patients in spite of higher incidence of grade ≥ 3 TRAEs. When stratified by PD‐L1 expression, patients with negative PD‐L1 expression can further benefit from combination therapy in mOS, mPFS, and ORR, regardless of atezolizumab or pembrolizumab. However, patients with low PD‐L1 expression (1% ≤ PD‐L1 ≤ 49%) show benefits in mOS and OSR1y only from chemotherapy in combination with pembrolizumab instead of atezolizumab, in spite of a significant improved PFS. Interaction tests showed that pembrolizumab combined with chemotherapy significantly reduce the mortality rate vs ICIs alone for the patients with PD‐L1 low expression. In summary, ICIs combined with chemotherapy is a favorable treatment choice for advanced NSCLC with low or negative PD‐L1 expression due to significant survival benefit. Sensitivity analysis showed that IMPOWER‐131 had a significant impact on OS results in patients with PD‐L1 low expression. Although the possible influencing factors such as pathological types, inclusion criteria of patients, treatment crossover and other factors have been taken into account and in fact they were well‐balanced, it is hard to explain the reason of increased mortality in chemotherapy combined with ICIs group compared with chemotherapy alone group.

We should interpret carefully which ICIs would be better when used in combination with chemotherapy because of no RCTs designed for direct comparison. Interaction test showed that pembrolizumab combined with chemotherapy brought more benefits vs atezolizumab combined with chemotherapy in mOS, mPFS, and ORR in PD‐L1 unselected patients. These results are consistent with previous study.^30^, ^31^ Possible explanation is that anti‐PD‐L1 antibody does not interfere the interaction between PD‐1 and PD‐L2.^32^, ^33^ Head‐to‐head clinical trials are needed for optimal drug selection.

Subgroup analysis based on histopathological type indicated that chemotherapy combined with ICIs, whatever atezolizumab or pembrolizumab, showed similar benefits in terms of mOS and mPFS between nonsquamous and squamous cell carcinoma in PD‐L1 unselected patients. In contrast, a better ORR was observed in nonsquamous cell carcinoma vs squamous cell carcinoma (all *P* < 0.05 for interaction).

Detection of PD‐L1 protein levels prior to treatment has been recommended by the NCCN guidelines for predicting the efficacy of pembrolizumab to make treatment choices. However, the current problem of detecting PD‐L1 involves lower sensitivity and specificity of efficacy prediction, and different IHC antibodies and test platforms, as well as inconsistent evaluation methods were used in different drug clinical studies.^34^ Recent studies demonstrated the percentage of PD‐L1‐stained tumor cells was highly comparable among 22C3, 28‐8, and SP263 PD‐L1 assays, while SP142 assay exhibited fewer stained tumor cells overall.^35^, ^36^ Considering that this might have an impact on the assessment of PD‐L1 levels, subgroup analysis for different ICIs was conducted when stratified by PD‐L1 level.

Previous studies have shown that the use of PD‐L1 expression status alone is insufficient to determine which patients should be offered PD‐1 or PD‐L1 blockade therapy^37^ and TMB might serve as a complementary diagnostic tool.^38^ High TMB is related with increased tumor‐infiltrating lymphocytes, expression of proinflammatory cytokines and immune‐related genes, but the impact on clinical outcome has yet to be clarified.^39^ For the patients with high TMB, the nivolumab monotherapy or in combination with ipilimumab showed no obvious difference in terms of OS compared with chemotherapy alone, while the combination of nivolumab and ipilimumab showed benefit in PFS and ORR. Many patients with a high TMB in the chemotherapy arm received subsequent nivolumab might influence the OS outcomes.^40^ However, the ability of TMB to predict the efficacy of ICIs may be insufficient and more clinical trials are needed to confirm it.

The test of TMB faces similar difficulties to PD‐L1 that methods for evaluating TMB in different laboratories have not yet been uniform, repeatability and optimal cutoff value have not yet determined. Different detection methods are needed to reach a unified standard and different standards for tissue biopsy samples and blood samples need to be further specified. Other biomarkers such as microsatellite instability‐high, mismatch repair‐deficient,^41^ and exosomal PD‐L1^42^ might be of guiding significance to predict efficacy. Therefore, further research with more accurate biomarkers is needed to guide the treatment of ICIs.

There are several limitations in this study. Firstly, this meta‐analysis was based on study‐level evidence, although the conclusions derived from the pooled data of eleven RCTs, different inclusion and exclusion criteria and the heterogeneity between studies should be noticed. Additionally, the secondary analyses based on the status of PDL1 expression and TMB mostly belong to retrospective analyses, and there might exist unbalanced factors between the study arms and the control arms. Moreover, in the group of ICIs combined with chemotherapies, different chemotherapy regimens, and anti‐angiogenic drugs might have different effects on new antigens and immune microenvironment and the effects of different drugs need further exploration. Furthermore, the test of interaction is an indirect comparison analysis, which might compromise the evidence level.

## 5 CONCLUSION

This study has demonstrated that ICIs in combination with chemotherapy significantly improve the disease control and reduce the risk of disease progression but have no statistically significant survival benefits compared with ICIs alone in the first‐line treatment of PD‐L1 high expression (TPS ≥ 50%) advanced NSCLC. The findings support the rationale for using pembrolizumab alone in the treatment of advanced NSCLC with PD‐L1 ≥ 50%. However, a significant survival benefit compared with chemotherapy alone even in PD‐L1 low or negative expression advanced NSCLC was observed in combination therapy, but it was associated with a higher incidence of grade ≥ 3 TRAEs.

## CONFLICT OF INTEREST

The authors declare no conflict of interest.

## AUTHOR CONTRIBUTIONS

RC and SZ conceived and designed the study. RC, LS, and XZ took full responsibility for data collecting. RC, YL, WJ, and LH performed the meta‐analysis, systematic review, and drafted the manuscript. CH and JM helped revise the manuscript.
