# Supplementary figures and images for: Rational application of the first‐line chemotherapy and immune checkpoint inhibitors in advanced nonsmall cell lung cancer: A meta‐analysis
Source: Cancer Med. 2019 Jul 11;8(11):5033–46. doi: 10.1002/cam4.2407 (PMC6718602; doi:10.1002/cam4.2407)

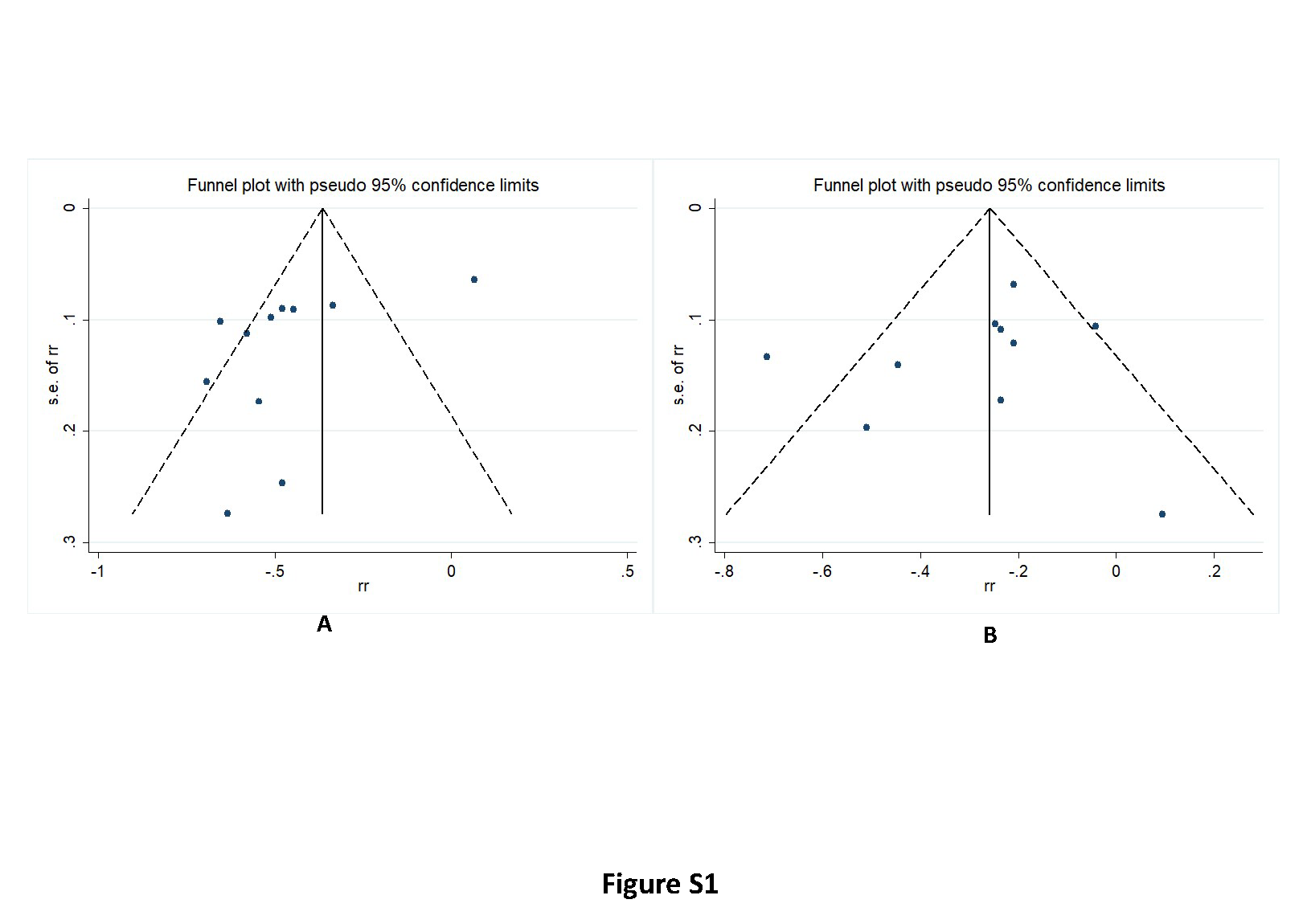

Supplement: Supplementary file 1 [file CAM4-8-5033-s001.tif]

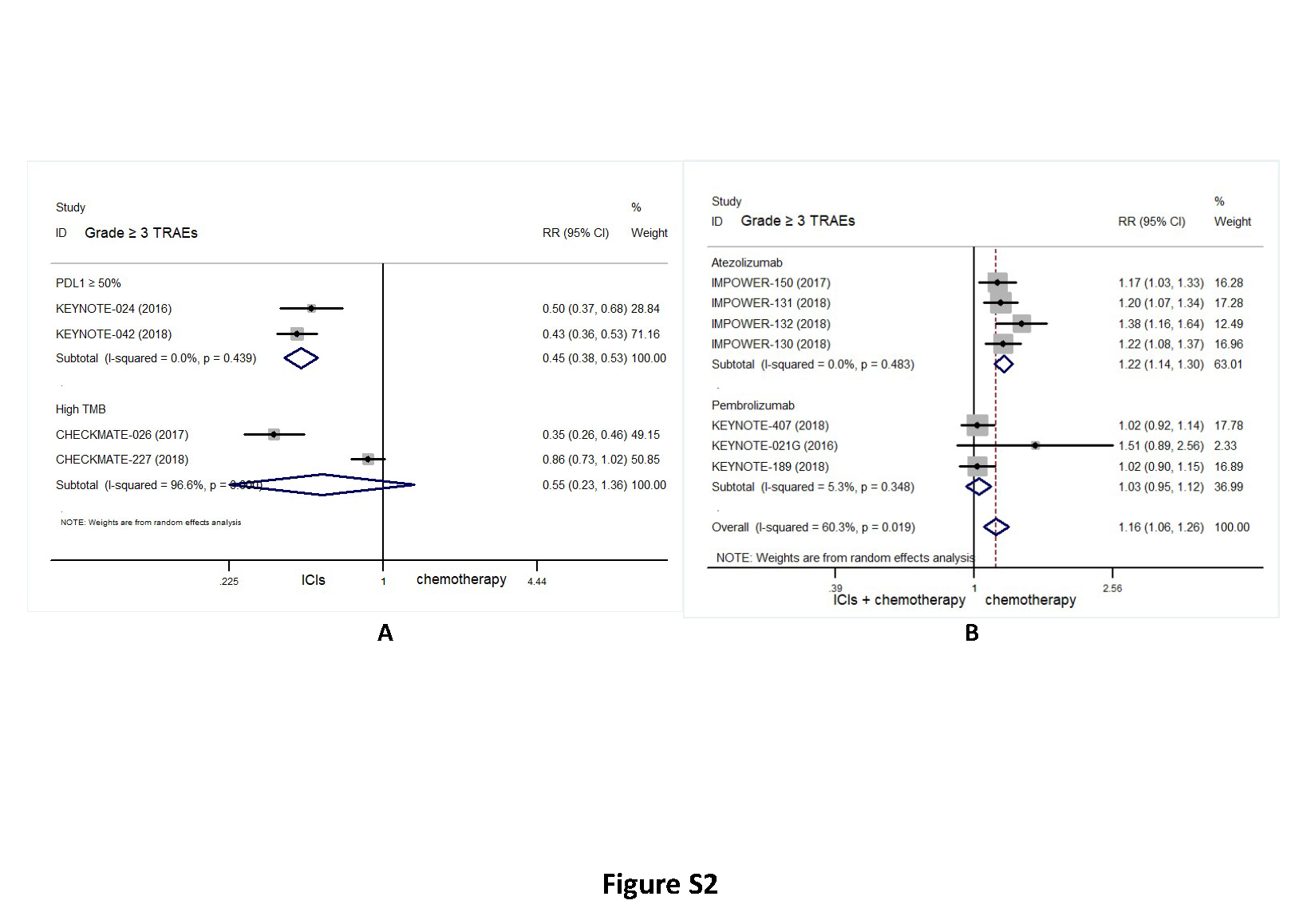

Supplement: Supplementary file 2 [file CAM4-8-5033-s002.tif]

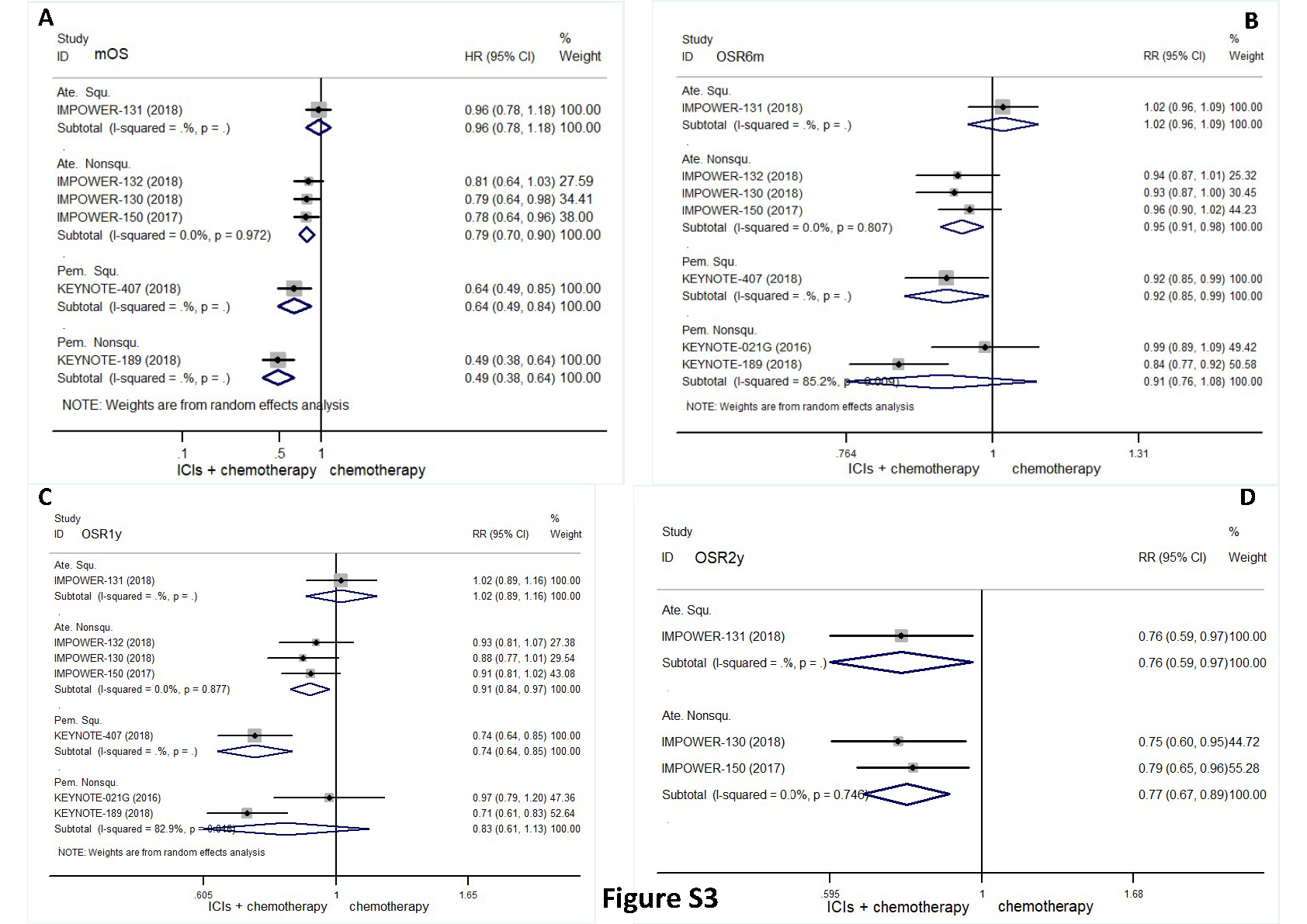

Supplement: Supplementary file 3 [file CAM4-8-5033-s003.tif]

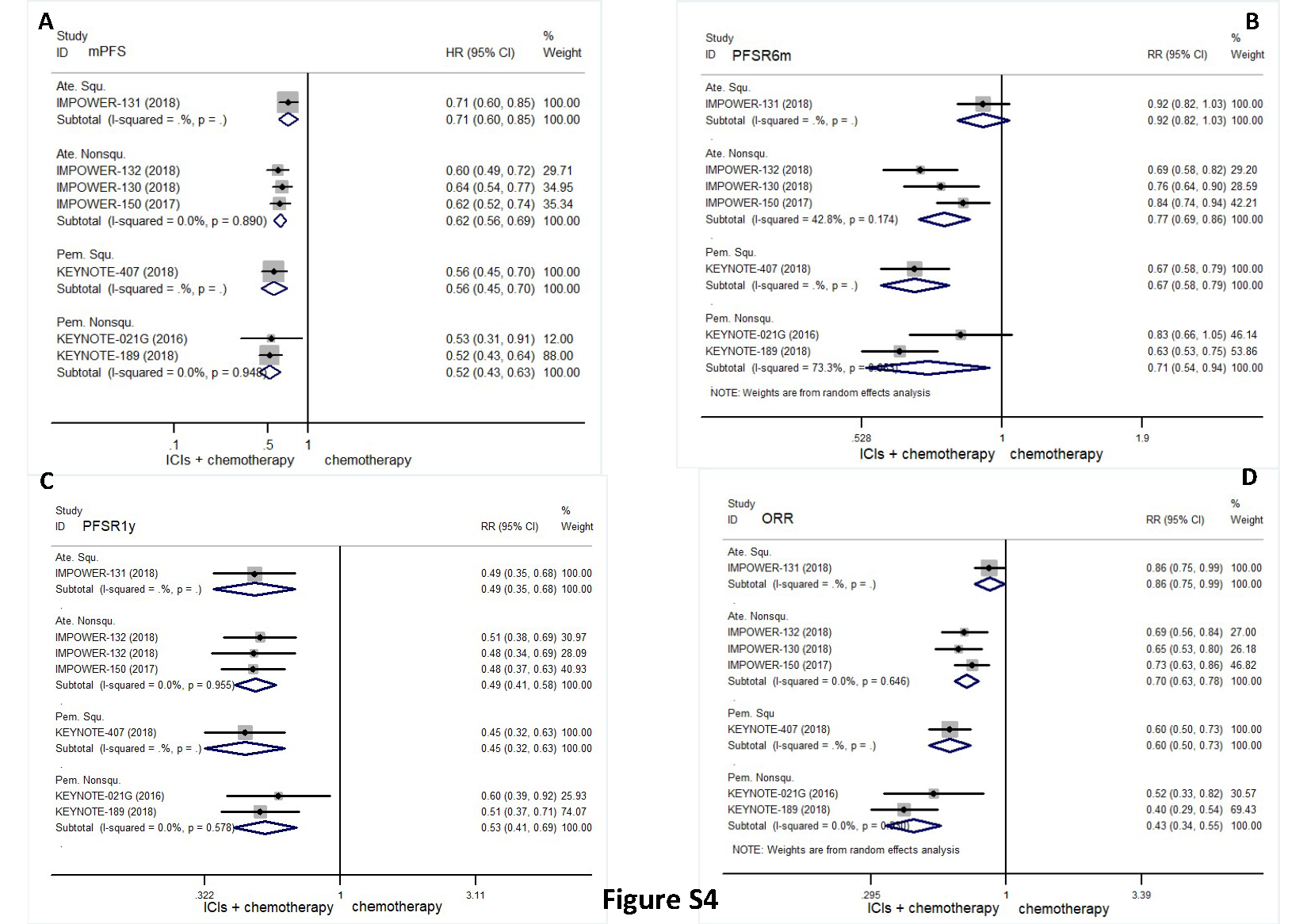

Supplement: Supplementary file 4 [file CAM4-8-5033-s004.tif]

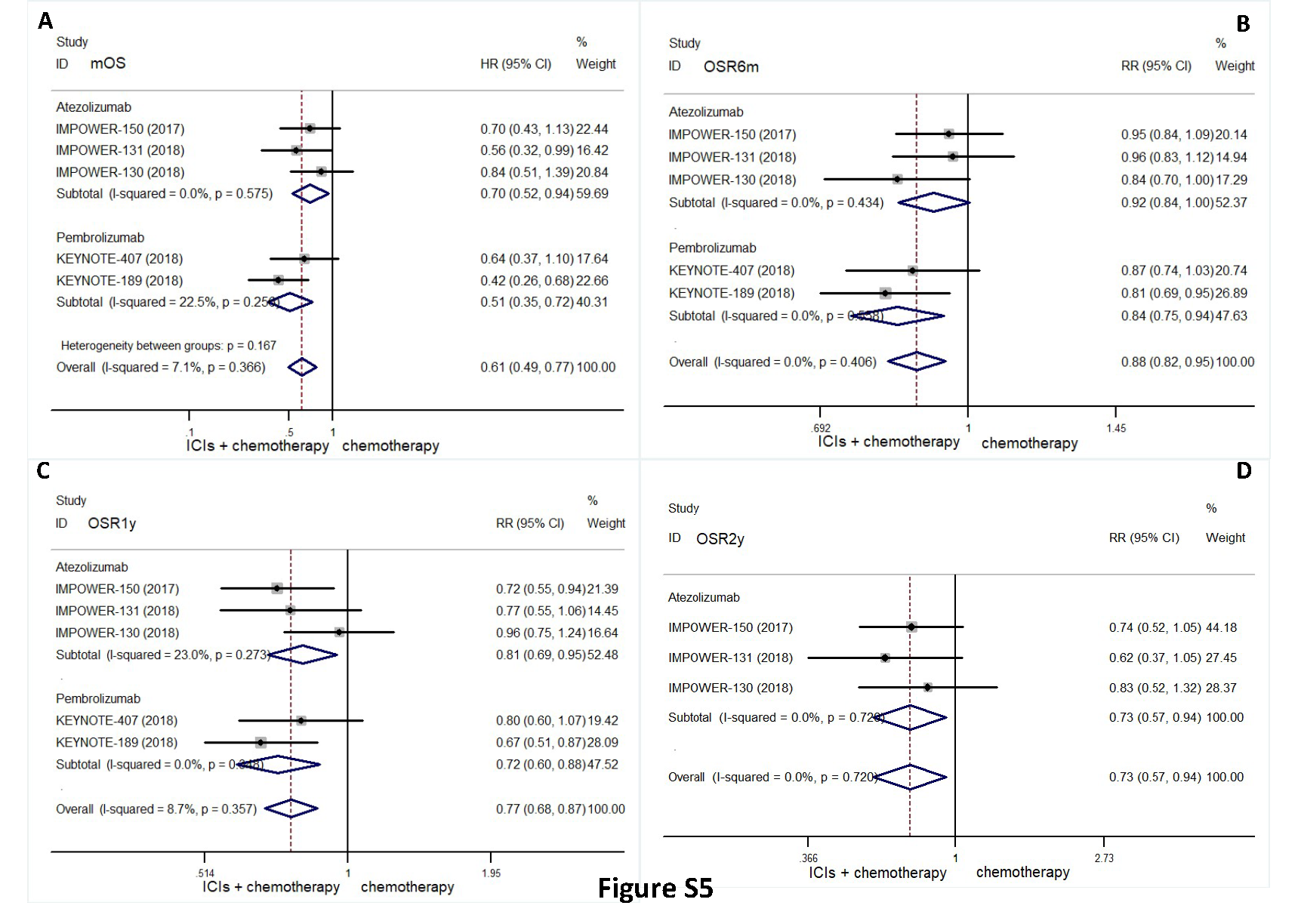

Supplement: Supplementary file 5 [file CAM4-8-5033-s005.tif]

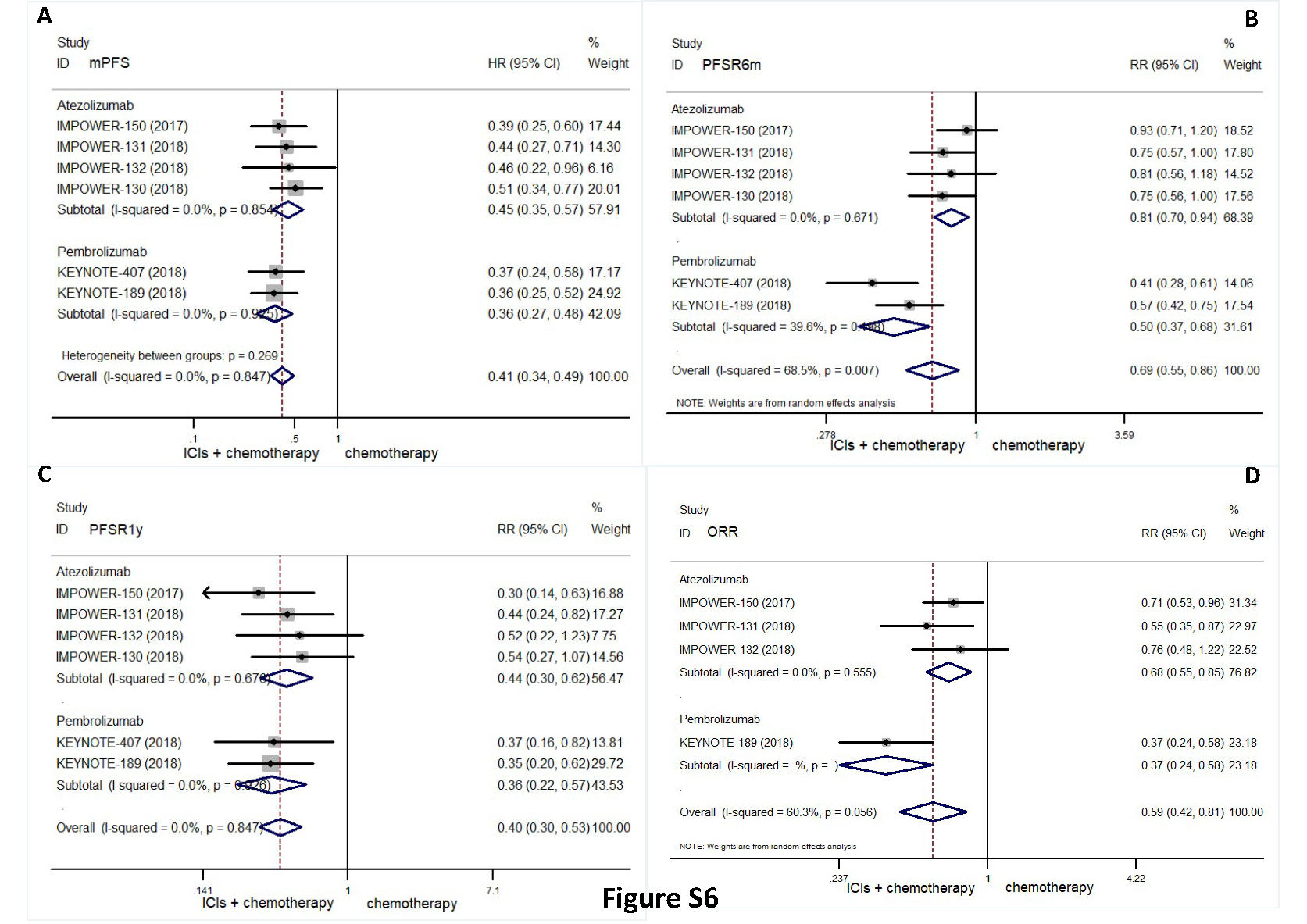

Supplement: Supplementary file 6 [file CAM4-8-5033-s006.tif]

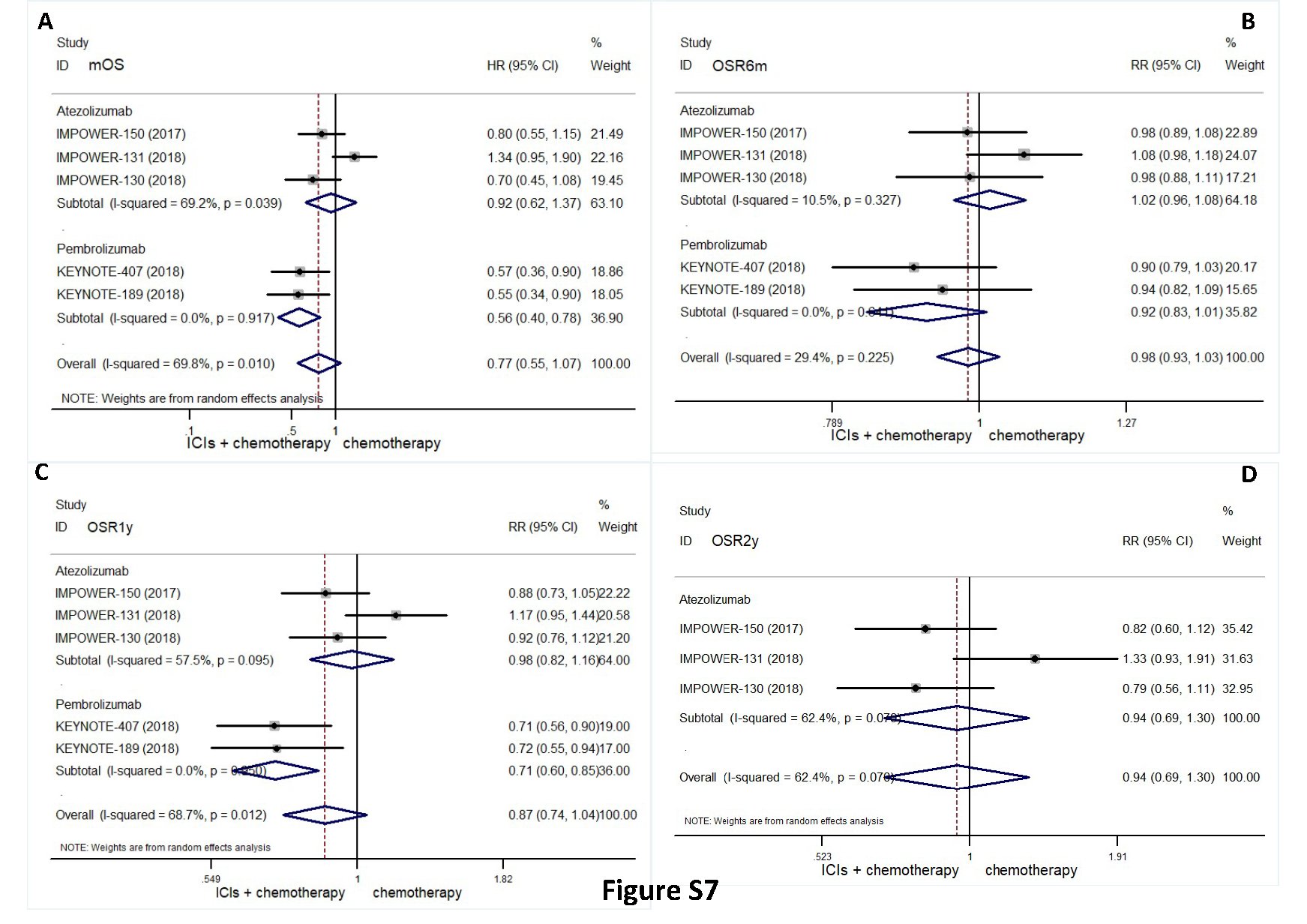

Supplement: Supplementary file 7 [file CAM4-8-5033-s007.tif]

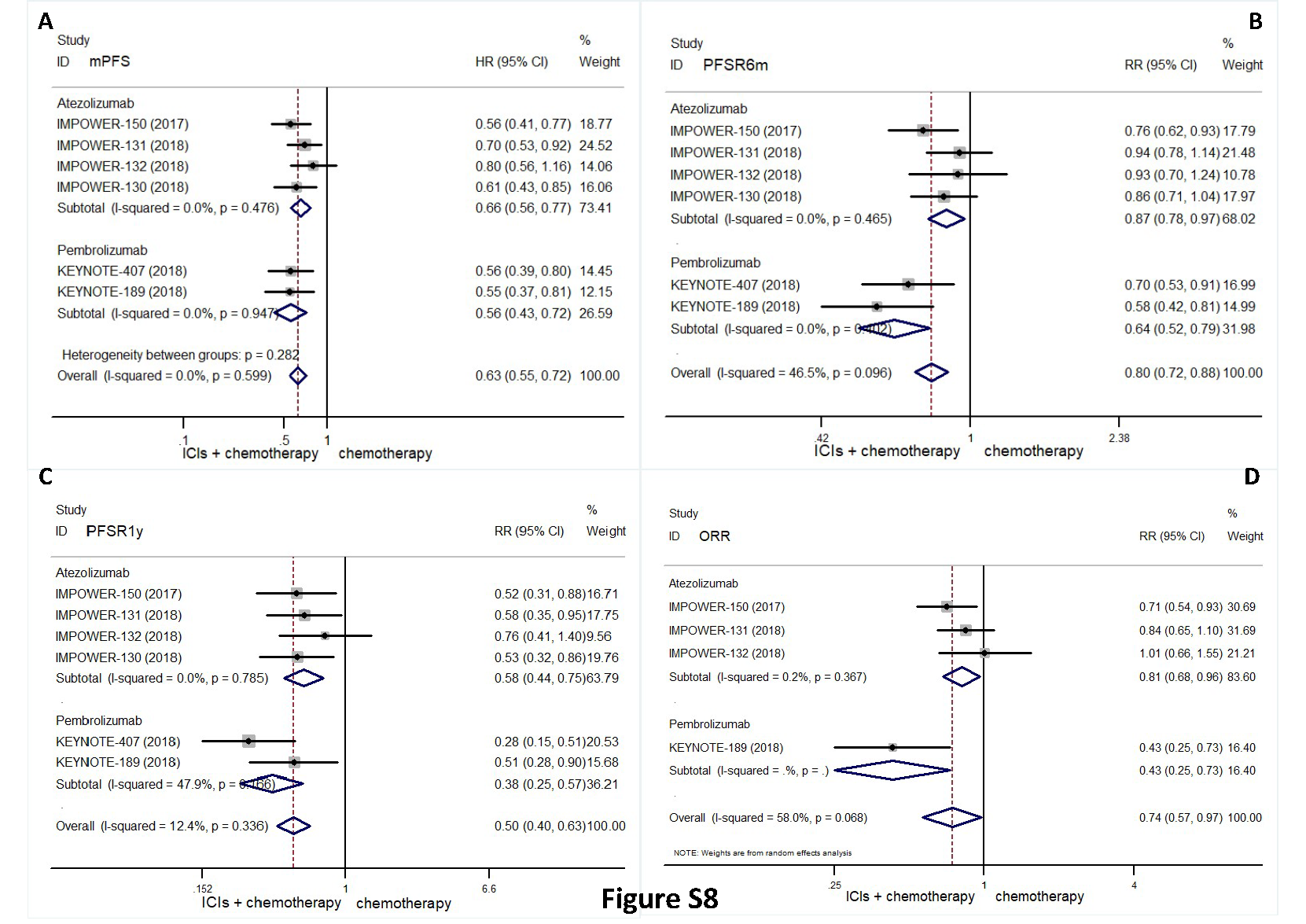

Supplement: Supplementary file 8 [file CAM4-8-5033-s008.tif]

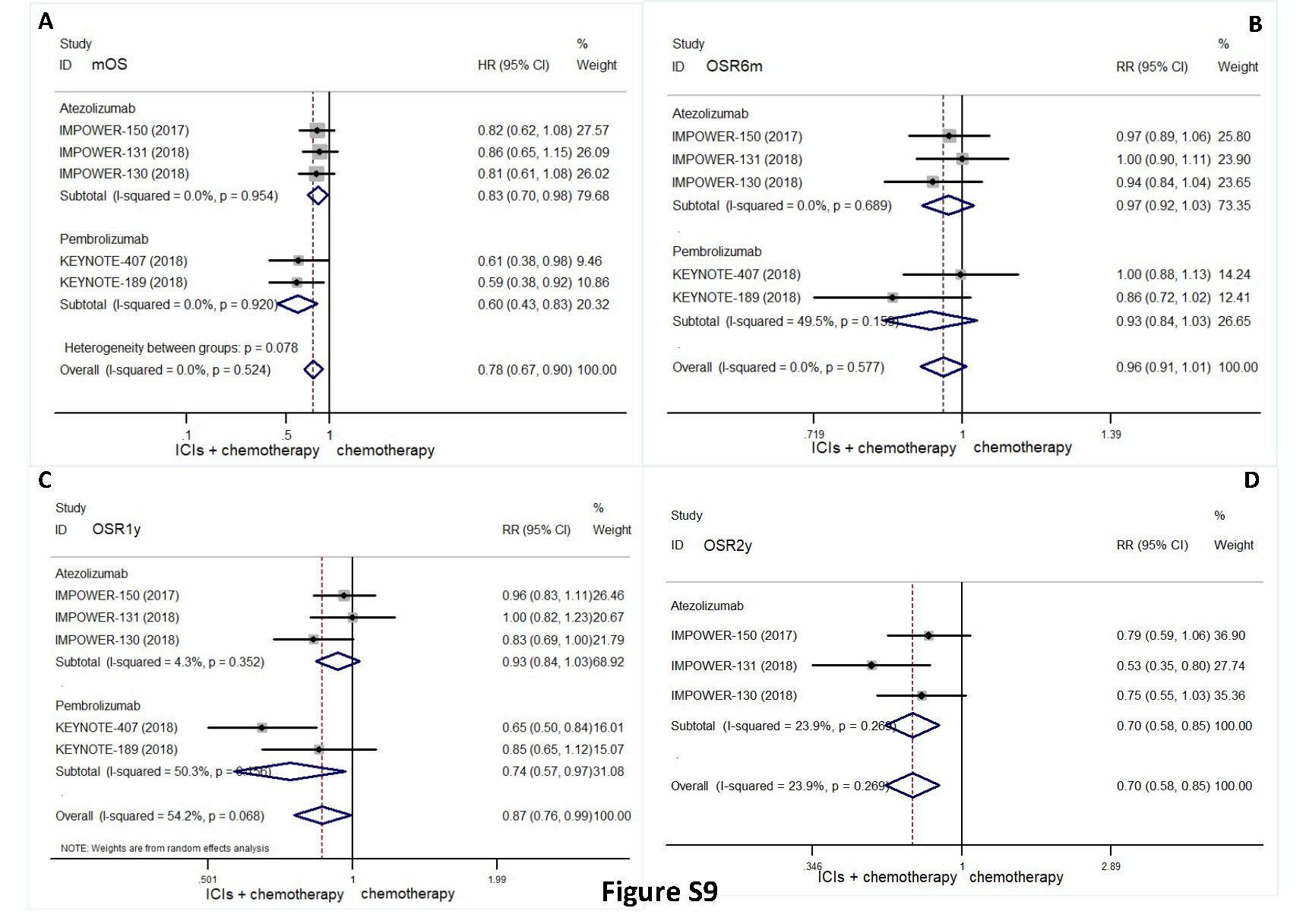

Supplement: Supplementary file 9 [file CAM4-8-5033-s009.tif]

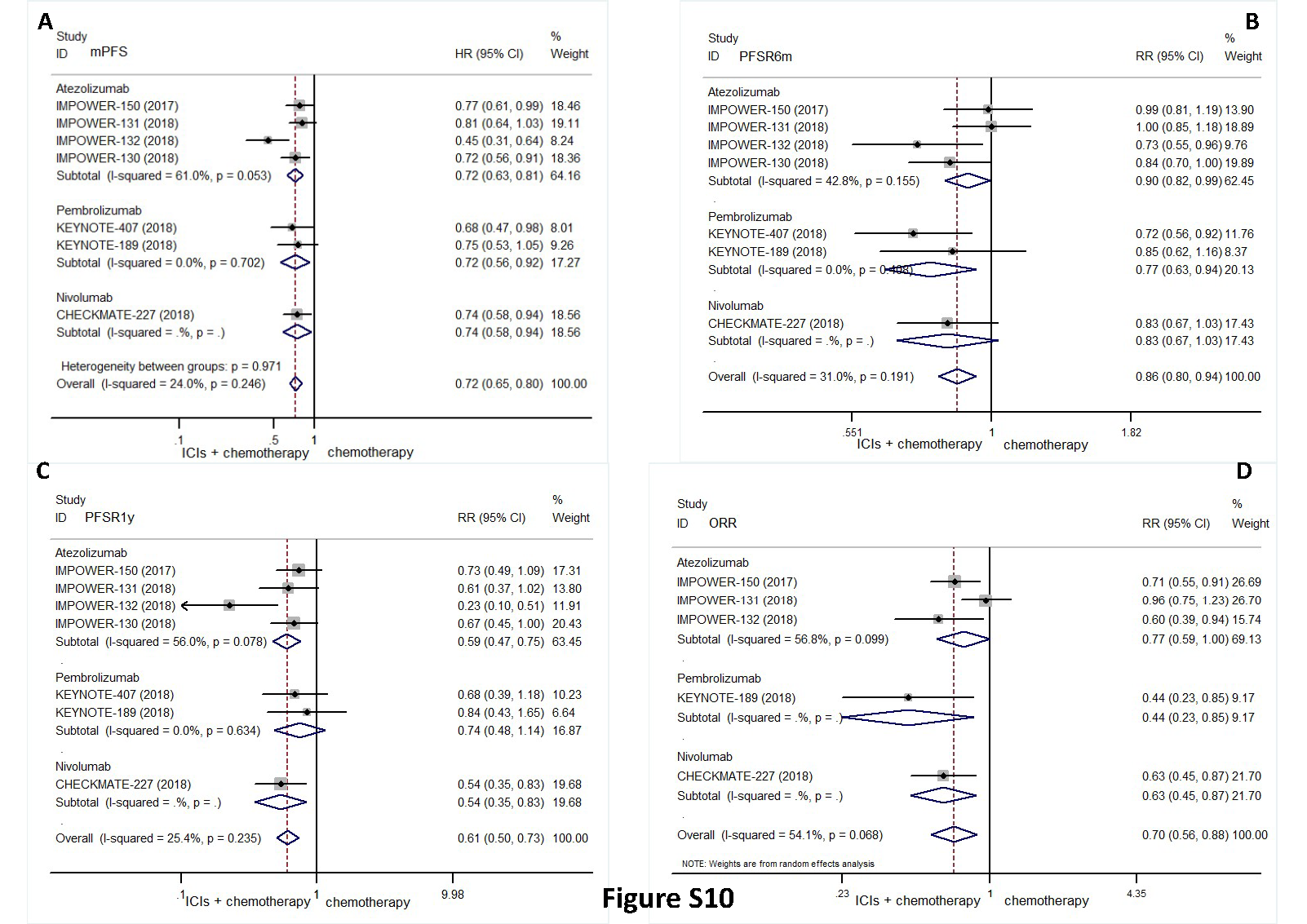

Supplement: Supplementary file 10 [file CAM4-8-5033-s010.tif]

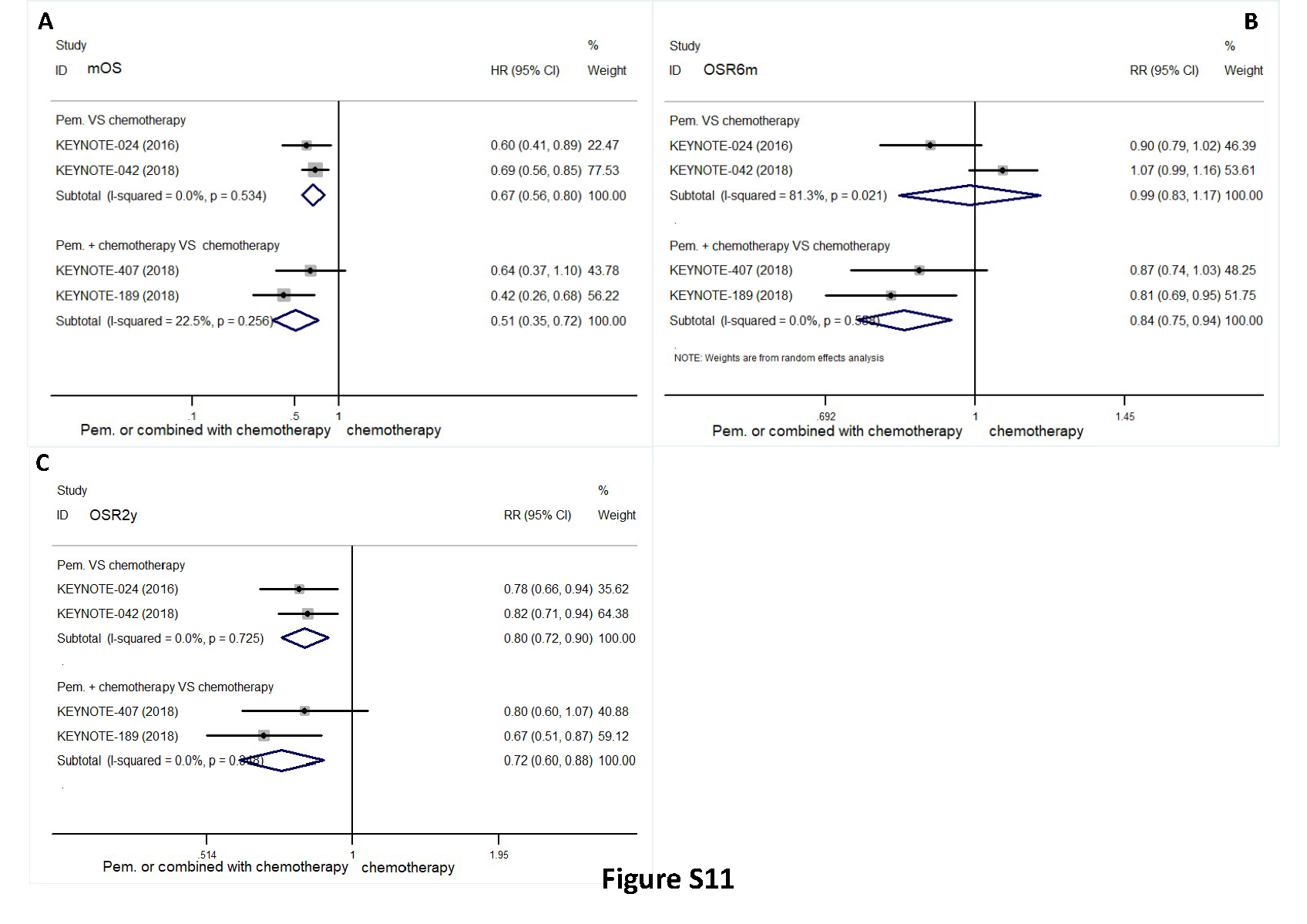

Supplement: Supplementary file 11 [file CAM4-8-5033-s011.tif]

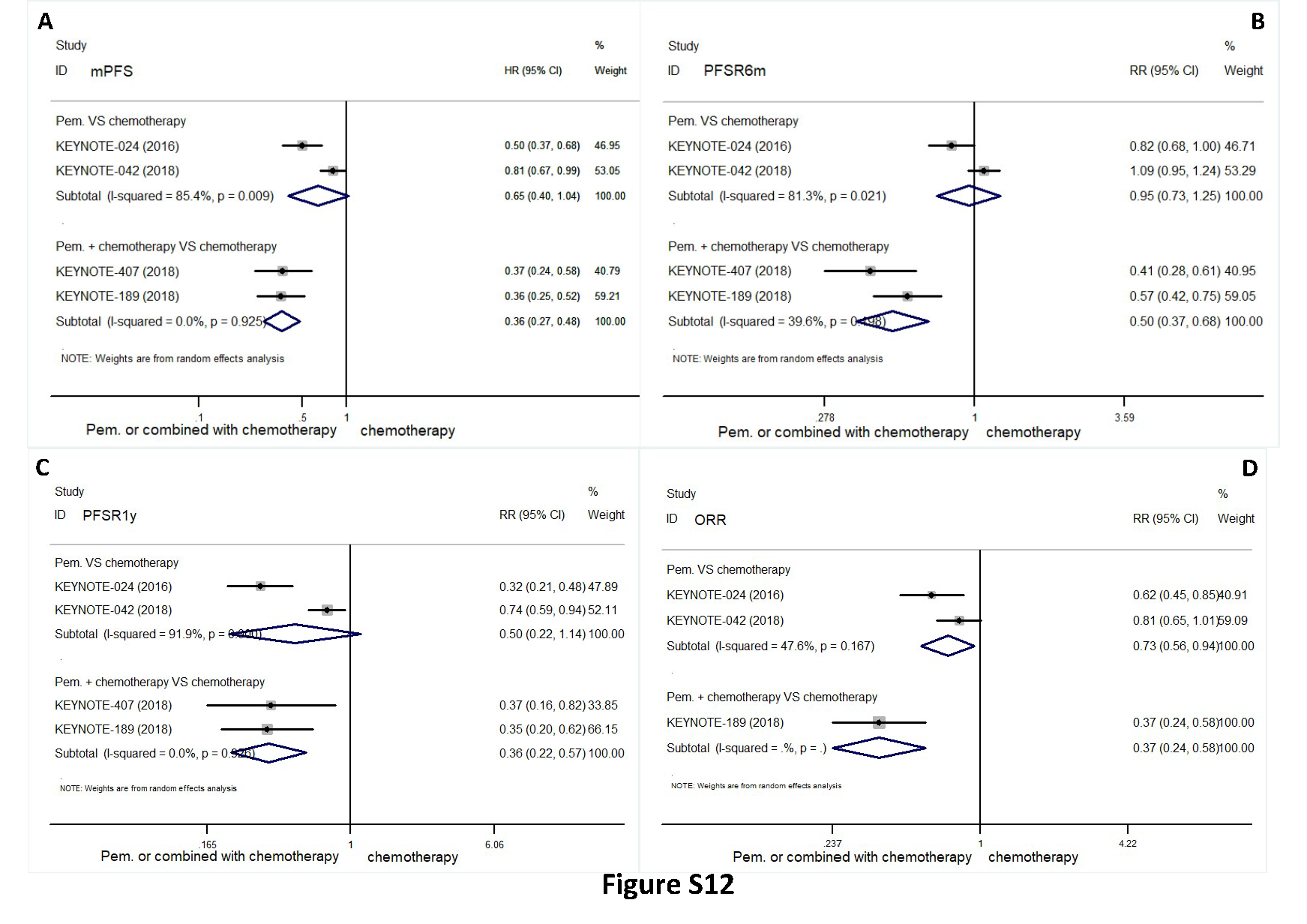

Supplement: Supplementary file 12 [file CAM4-8-5033-s012.tif]

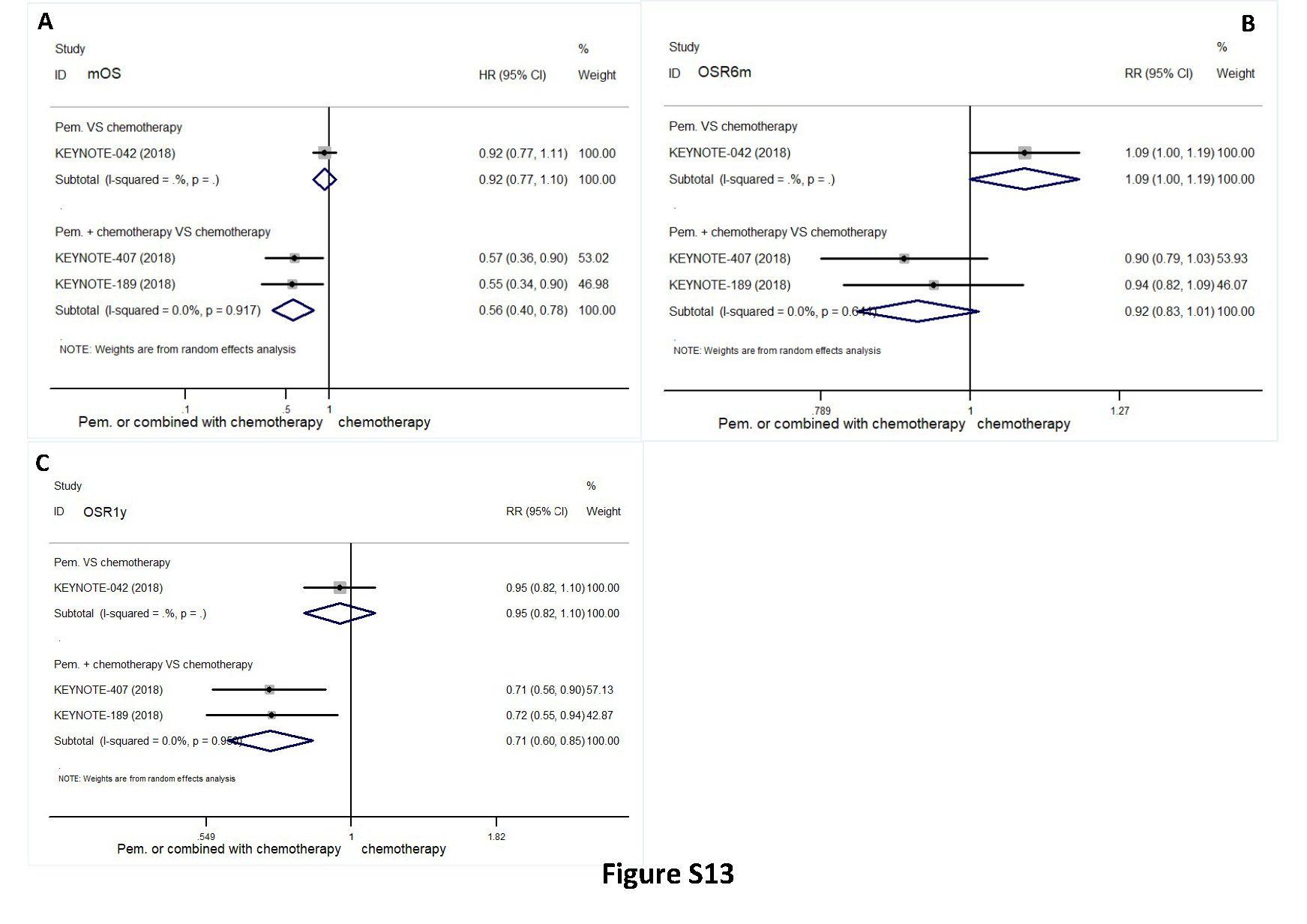

Supplement: Supplementary file 13 [file CAM4-8-5033-s013.tif]
